# *Notch* Missense Mutations in *Drosophila* Reveal Functions of Specific EGF-like Repeats in Notch Folding, Trafficking, and Signaling

**DOI:** 10.3390/biom12121752

**Published:** 2022-11-25

**Authors:** Hilman Nurmahdi, Mao Hasegawa, Elzava Yuslimatin Mujizah, Takeshi Sasamura, Mikiko Inaki, Shinya Yamamoto, Tomoko Yamakawa, Kenji Matsuno

**Affiliations:** 1 Department of Biological Science, Graduate School of Science, Osaka University, Toyonaka 560-0043, Japan; 2 Department of Molecular and Human Genetics, Baylor College of Medicine, Houston, TX 77030, USA; 3 Jan and Dan Duncan Neurological Research Institute, Texas Children’s Hospital, Houston, TX 77030, USA

**Keywords:** Notch, Notch signaling pathway, lateral inhibition, asymmetric cell division, protein folding, intracellular trafficking, endoplasmic reticulum, neurogenic phenotype, hindgut, *Drosophila*

## Abstract

Notch signaling plays various roles in cell-fate specification through direct cell–cell interactions. Notch receptors are evolutionarily conserved transmembrane proteins with multiple epidermal growth factor (EGF)-like repeats. *Drosophila* Notch has 36 EGF-like repeats, and while some play a role in Notch signaling, the specific functions of most remain unclear. To investigate the role of each EGF-like repeat, we used 19 previously identified missense mutations of *Notch* with unique amino acid substitutions in various EGF-like repeats and a transmembrane domain; 17 of these were identified through a single genetic screen. We assessed these mutants’ phenotypes in the nervous system and hindgut during embryogenesis, and found that 10 of the 19 *Notch* mutants had defects in both lateral inhibition and inductive Notch signaling, showing context dependency. Of these 10 mutants, six accumulated Notch in the endoplasmic reticulum (ER), and these six were located in EGF-like repeats 8–10 or 25. Mutations with cysteine substitutions were not always coupled with ER accumulation. This suggests that certain EGF-like repeats may be particularly susceptible to structural perturbation, resulting in a misfolded and inactive Notch product that accumulates in the ER. Thus, we propose that these EGF-like repeats may be integral to Notch folding.

## 1. Introduction

Cell signaling is essential in the regulation of various biological processes. Notch signaling plays crucial roles in development and homeostasis across phyla [1,2], and regulates cell-fate specification, cell physiology, apoptosis, and pattern formation through direct cell–cell interactions [3]. Components of the Notch signaling pathway are evolutionarily conserved, and studies of Notch signaling in invertebrate model organisms such as *Drosophila melanogaster* have contributed significantly to a mechanistic understanding of how the pathway works in vertebrates [1,2]. In humans, Notch signaling plays vital roles in development and homeostasis, and aberrant Notch signaling causes various diseases [4]. The major steps of the Notch signaling cascade have been revealed through genetic, biochemical, and cell biology studies [3]. Notch receptor and its ligands, designated as DSL (Delta/Serrate/Lag-2) ligands, are single-pass transmembrane proteins that are synthesized in the endoplasmic reticulum (ER) and trafficked to the cell surface through the Golgi complex [5]. When DSL ligands presented by a signaling cell bind the extracellular domain of Notch on the signal-receiving cell, Notch undergoes two successive proteolytic cleavages by ADAM-family metalloproteases and the γ-secretase complex [6]. Consequently, the Notch intracellular domain (NICD) is freed from the plasma membrane and translocated into the nucleus, where NICD forms a complex with CSL (CBF1/Suppressor of Hairless/Lag-1) transcription factor and promotes the transcription of target genes [6,7,8].

**Figure 1:**
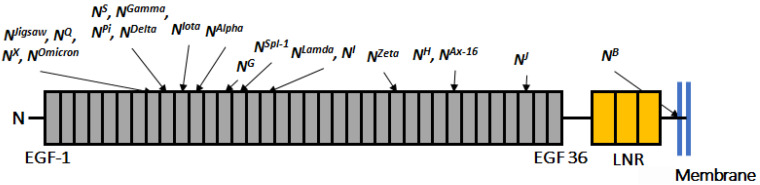
A schematic diagram of the Notch extracellular domain and the positions of EGF-like repeats with amino acid substitutions in *Notch* mutations analyzed in this study. The extracellular domain of *Drosophila* Notch has 36 epidermal growth factor (EGF)-like repeats (gray), three lin12/Notch repeats (LNR) (orange), and a transmembrane domain (blue lines). We investigated 19 missense mutations of *Notch* with amino acid substitutions in EGF-like repeats and the transmembrane domain. The mutant alleles are listed across the top, with arrows pointing to the position of the EGF-like repeat and the transmembrane domain with the substitution. EGF-like repeats are numbered in order starting with the repeat closest to the N-terminal (EGF-1).

The extracellular domains of *Drosophila* Notch and mammalian Notch-1 and Notch-2 receptors have 36 EGF-like repeats that serve as sites for *cis*- and *trans*-interactions with ligands [9]. An EGF-like repeat generally consists of ~40 amino acid residues that mostly form two β-sheet structures and contain six conserved cysteines (C1-C6) forming three disulfide bonds in the following interactions: C1-C3, C2-C4, and C5-C6 [10,11,12,13,14]. Regardless of similarities in sequence and structure in the EGF-like repeats, each repeat likely plays its own distinct roles in ligand–receptor interactions and signaling. For example, among the 36 EGF-like repeats of *Drosophila* Notch and mammalian Notch-1, an EGF-like repeat of any number (assigned by its position in the sequence of EGF-like repeats, counted out from the N-terminal) is most similar to the corresponding EGF-like repeats of the other Notch paralogs, compared with any other EGF-like repeat located in a different region of the same protein [2]. This observation suggests that the alignment sequence of the EGF-like repeats is important and that each EGF-like repeat has position-specific roles. In fact, EGF-like repeats 11–12 were identified as the core ligand-binding site (Figure 1) [15]. More recently, studies involving structural biology revealed that EGF-like repeats 11–12 and 8–12 interface with the ligands Delta-like 4 and Jagged1, respectively [15,16].

Protein glycosylation adds another layer of specificity to EGF-like repeats in Notch signaling because of specific glycan modifications present in various EGF-like repeats, including *O*-fucosylation, *O*-glucosylation, and *N*-glycosylation [17]. These glycan modifications have unique and redundant roles in Notch signaling. For example, *O*-fucose glycan added to EGF-like repeats in the Notch ligand-binding site (EGF-like repeats 11–12) directly contributes to ligand–receptor interactions, as it lies within the binding pocket and modulates the specificity of the interaction between Notch and the two ligand types, Delta and Serrate/Jagged [15,16]. Although about two thirds of EGF-like repeats have some of these *O*-fucose glycan modifications, the modifications are important only for specific EGF-like repeats, such as EGF-like 6, 8, 9, 12, and 36, in regulating Notch–ligand interactions [18,19]. Thus, individual EGF-like repeats with *O*-fucose glycan modifications play specific roles. In addition, we previously reported that *O*-fucose and *O*-glucose glycans have redundant functions in folding Notch in vivo [20]. In our previous study of *Drosophila* missense mutations in Notch EGF-like repeats*,* we revealed that Notch accumulates in the ER when *O*-fucose and *O*-glucose glycans are simultaneously removed, but not when either glycan alone is depleted [20]. Since some of the EGF-like repeats lack the modification sites for these *O*-glycans, each EGF-like repeat likely differs in its response to the structural perturbation induced by depleting these glycans. The hypothesis that different EGF-like repeats on Notch receptors have specific functions is also supported by genetic evidence [21,22,23]. For example, a class of *Notch* gain-of-function alleles, called *Abruptex* mutants, were found to carry missense mutations introducing amino acid substitutions in EGF-like repeats 24–29, designated as the Abruptex domain [21]. Therefore, it has been suggested that EGF-like repeats 24–29 negatively regulate the Notch receptor [21,22,23]. However, the specific roles of most of the EGF-like repeats or clusters of repeats in Notch signaling are not clear.

A previous study obtained a collection of new *Notch* mutants isolated on an isogenized chromosome through a forward genetic screen based on lethality and morphological phenotypes in the wing and mechanosensory bristles (Figure 1) [24]. Sixteen *Notch* mutant alleles isolated from this single genetic screen carry unique single amino acid substitutions in the different EGF-like repeats, which gave us an opportunity to investigate specific functions of the individual EGF-like repeats. Notch signaling is known to contribute to three conceptually and molecularly distinct classes of signaling events: lateral inhibition, inductive signaling, and asymmetric cell division [3]. Phenotypes associated with the wing margin and wing veins were screened to isolate these mutants through clonal analysis; these phenotypes are associated with the disruption of *Notch* activity in inductive signaling and lateral inhibition during larval development [25,26,27,28]. Morphological defects in mechanosensory bristle, screened in the dorsal thorax, often reflect defects in lateral inhibition and asymmetric cell division of the peripheral nervous system during pupal development [24,27,28].

In the present study, we also considered phenotypes associated with defects in Notch signaling in neurogenesis and hindgut development. Neural hyperplasia of the embryonic central nervous system, a neurogenic phenotype, occurs when Notch signaling activity is depleted in lateral inhibition during early embryogenesis, since wild-type Notch signaling restricts the number of neuroblasts through lateral inhibition in the developing neuroectoderm [29]. In later embryogenesis, Notch is important for patterning the digestive tract through inductive signaling, which can be analyzed by observing the formation of boundary cells between the dorsal and ventral compartments of the hindgut epithelium in embryos [30]. The hindgut epithelium is also useful for analyzing intracellular Notch localization [31], since wild-type Notch mostly localizes to adherens junctions (AJs) in the epithelium of the embryonic hindgut and other epithelia [31,32], but defective Notch folding causes Notch to accumulate in the ER instead of the AJs [33]. Aberrant vesicular Notch trafficking and endocytosis can be assessed by Notch accumulation in endocytic compartments of various epithelial tissues [20,33,34,35]. Therefore, the epithelium of the embryonic hindgut was useful for studying defective Notch folding and trafficking in the *Notch* mutants.

Here, we analyzed *Notch* mutant alleles isolated through the genetic screening noted earlier, along with two classic *Notch* mutants that affect EGF-like repeats, and systematically investigated their effects on Notch signaling activity during lateral inhibition in the developing embryonic central nervous system and inductive signaling in the embryonic hindgut. We found that EGF-like repeats with different missense mutations produce specific defects in the activity, folding, and trafficking of Notch, and some mutations form a discrete cluster. These results suggest that each EGF-like repeat or cluster of repeats is specific in its contributions to Notch structure and function.

## 2. Materials and Methods

### 2.1. Drosophila Stocks and Crosses

All experiments were performed at 25 °C using standard *Drosophila* culture media. Canton-S was used as a wild-type control line. Our collection of *Notch* mutants with missense mutations that introduce an amino acid substitution to an EGF-like repeat has been described [24,27,28]. These *Notch* mutants, which are recessive lethal, were maintained on an *FM7c Kr > GFP* balancer chromosome [24,27,28].

We used the following *Notch* mutants from our collection: *N^X^* (DGRC 116669), *N^Omicron^* (DGRC 116715), *N^jigsaw^* (DGRC 116622), *N^Gamma^* (DGRC 116750), *N^S^* (DGRC 116605), *N^Iota^* (DGRC 116608), *N^G^* (DGRC 116671), *N^I^* (DGRC 116689), *N^Zeta^* (DGRC 116597), *N^H^* (DGRC 116684), *N^J^* (DGRC 116700), *N^B^* (DGRC 116625), *N^Q^* (DGRC 116732), *N^Pi^* (DGRC 116764), *N^Delta^* (DGRC 116573), *N^Alpha^* [24] and *N^Lambda^* [24]. We also used the classic *Notch* alleles *N^spl−^*^1^ (BDSC 182), *N^Ax−^*^16^ (BDSC 52014), and *N*^55*e*11^ (BDSC 28813) in some experiments. We used a *Pdi-GFP* protein trap line (DGRC 110624) to detect protein disulfide isomerase (Pdi), a typical marker of the ER [36]. To observe Pdi-GFP in the epithelium of the embryonic hindgut, females heterozygous for each *Notch* mutant (*N^mutant^*/*FM7c Kr > GFP*) were crossed with males of *+/*Y; *Pdi-GFP/Pdi-GFP*. Male embryos hemizygous for each *Notch* mutant (*N^mutant^*/Y) were selected based on their neurogenic phenotype and the absence of *FM7c Kr > GFP*.

### 2.2. Immunostaining

Embryos were stained with antibodies as previously described [37] and observed with a Zeiss LSM 700 or LSM 810 confocal laser microscope, and the results were analyzed using the LSM image browser (Zeiss, Jena, Germany) and ImageJ software (Version 13.0.6, NIH, Bethesda, MD, USA) [38]. We used the following primary antibodies: rat anti-Elav (7E8A10, 1:500) [39], mouse anti-NICD (C17.9C6, 1:250) [40], mouse anti-Crumbs (Cq4, 1:250) [41], rat anti-E-Cadherin (DCAD2 1:500) [42], guinea pig anti-FL-Hrs (GP30, 1:1000) [43], rabbit anti-Rab7 (1:5000) [44], rabbit anti-Rab11 (1:4000) [44], rabbit anti-GM130 (1:50, Abcam, Cambridge, MA, USA) [45], rabbit anti-GFP (1:250, 598 MBL) [46], and rat anti-GFP (1:250, Nacalai Tesque, Kyoto, Japan).

We used the following secondary antibodies: Cy3-conjugated donkey anti-mouse, Cy5-conjugated anti-rabbit, Cy-5 conjugated anti-rat, Cy5-conjugated anti-guinea pig, Alexa488-conjugated donkey anti-rat, and Alexa488-conjugated donkey anti-rabbit (all from Jackson Immunoresearch, West Grove, PA, USA).

## 3. Results

### 3.1. Notch Missense Mutations That Affected the Development of the Embryonic Nervous System

We investigated the roles of individual EGF-like repeats in Notch signaling using a previously established collection of 17 missense mutations of *Notch* [24,27,28]. These mutants carry a single missense mutation, identified by DNA sequencing, in the *Notch* locus corresponding to the EGF-like repeats (*n* = 16) or in the transmembrane domain (*n* = 1) [24,27,28]; Figure 1 and Table 1 show the amino acid substitution, position, and EGF-like repeat affected for each mutant. We also included two classic missense alleles of *Notch*, *N^Spl−^*^1^ and *N^Ax−^*^16^, bringing the total number of *Notch* missense mutants examined in this study to 19.

**Figure 2:**
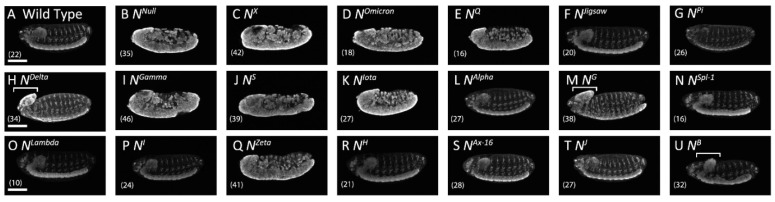
*Notch* alleles that disrupted lateral inhibition in the embryonic central nervous system. Lateral views show the embryonic nervous system, stained with an anti-Elav antibody (white), in (**A**) wild-type *Drosophila* and hemizygotes for (**B**) *N*^55*e*11^, an amorphic allele of *Notch*; (**C**) *N^X^*, (**D**) *N^Omicron^*, (**E**) *N^Q^*, (**F**) *N^Jigsaw^*, (**G**) *N^pi^*, (**H**) *N^Delta^*, (**I**) *N^Gamma^*, (**J**) *N^S^*, (**K**) *N^Iota^*, (**L**) *N^Alpha^*, (**M**) *N^G^*, (**N**) *N^Spl−^*^1^, (**O**) *N^Lambda^,* (**P**) *N^I^*, (**Q**) *N^Zeta^*, (**R**) *N^H^*, (**S**) *N^Ax^*^−16^, (**T**) *N^J^*, and (**U**) *N^B^*. White brackets show the regions with a brain deformation phenotype. The number of embryos analyzed is shown in parentheses. Scale bars: 100 μm.

In the embryonic central nervous system, depleted Notch signaling causes neural hyperplasia, designated as a neurogenic phenotype [47]. In this study, we observed neuronal cells by immunostaining with an antibody against the neuron-specific nuclear protein Elav (embryonic lethal abnormal vision) [48]. In wild-type embryos, anti-Elav stained the neuronal nuclei of the ladder-like nervous system (Figure 2A); in contrast, the classic *Notch* amorphic (null) allele *Notch*^55*e*11^ produced a severe neurogenic phenotype, with nearly the entire embryo stained by anti-Elav (Figure 2B) [47]. Of the 19 *Notch* alleles tested, each carrying a different missense mutation (Figure 2), 10 had a neurogenic or brain deformation phenotype (Figure 2C–E,H–K,M,Q,U; Table 1). Although the nature of brain deformation phenotype remained unclear, intensity of anti-Elav staining increased in these deformed brains, suggesting their neural hyperplasia that implies region-specific reduction of Notch signaling (Figure 2H,M,U; Table 1). The remaining nine mutants exhibited a wild-type nervous system, even though the same alleles produced a Notch signaling-related phenotype in other contexts (Figure 2F,G,L,N–T; Table 1) [24]. Considering that the role of Notch signaling is context-dependent in various tissues and organs [24,49], these missense mutations likely disrupt Notch signaling in a context-dependent manner.

### 3.2. Missense Notch Mutations That Affected Boundary Cell Formation in the Hindgut

We next examined these *Notch* mutants for defects in boundary cells in the embryonic digestive system, since boundary cell formation is a typical example of inductive Notch signaling [30,50]. The expression of the ligand Delta is limited to the ventral compartment of the hindgut because *engrailed*, which suppresses *Delta* expression, is expressed in the dorsal compartment [30]. In the ventral cells where *Delta* is expressed, Notch signaling is suppressed in most cells by *cis*-inhibition of Notch via Delta [30]. However, since Delta presented from the ventral cells can signal Notch receptors expressed in the dorsal cells, where *cis*-inhibition does not take place, Notch signaling is activated in the single row of dorsal cells that subsequently differentiates into boundary cells [30].

**Figure 3:**
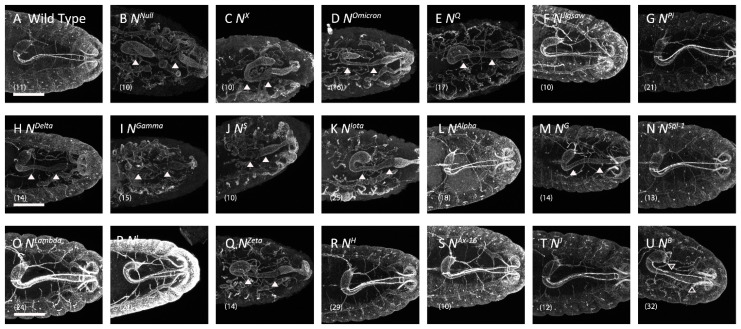
*Notch* alleles that induced defects in boundary cell formation. Dorsal views of the embryonic hindgut epithelium, stained with an anti-Crumbs antibody to detect boundary cells (white), in (**A**) wild-type embryos and the following hemizygotes: (**B**) *N*^55*e*11^, an amorphic allele of *Notch*; (**C**) *N^X^*, (**D**) *N^Omicron^*, (**E**) *N^Q^*, (**F**) *N^Jigsaw^*, (**G**) *N^pi^,* (**H**) *N^Delta^*, (**I**) *N^Gamma^*, (**J**) *N^S^*, (**K**) *N^Iota^*, (**L**) *N^Alpha^*, (**M**) *N^G^*, (**N**) *N^Spl−^*^1^, (**O**) *N^Lambda^*, (**P**) *N^I^*, (**Q**) *N^Zeta^*, (**R**) *N^H^*, (**S**) *N^Ax−^*^16^, (**T**) *N^J^*, and (**U**) *N^B^*. Filled white arrows indicate regions where anti-Crumbs antibody staining is depleted. Arrows outlined in white indicate regions where anti-Crumbs antibody staining showed abnormal gaps. The number of embryos analyzed is shown in parentheses. Scale bars: 50 μm.

Thus, we analyzed boundary cell formation to determine whether Notch signaling was disrupted in the *Notch* missense mutants during the development of the digestive system. The boundary cells highly express *crumbs*, which is required to establish apical-basal cell polarity and contributes to the organization of zonula adherens [51]. When stained with an anti-Crumbs antibody, boundary cells were observed as two narrow bands, each composed of a single row of boundary cells (Figure 3A) [51]. We confirmed that *crumbs* expression is lost in embryos hemizygous for *Notch*^55*e*11^ as previously described (Figure 3B), demonstrating that our assay has sufficient sensitivity for our purposes [30]. We assessed the presence or absence of boundary cells in embryos hemizygous for each *Notch* missense mutation and found that *crumbs* expression was depleted or showed abnormal gaps in 10 of the 19 *Notch* missense mutants (Figure 3C–E,H–K,M,Q,U; Table 1). However, the remaining nine missense mutations did not affect *crumbs* expression, indicating that inductive signaling was normal in this context (Figure 3F,G,L,N–P,R–T; Table 1) [24]. The 10 mutants with defective inductive signaling were the same 10 mutants with neurogenic or brain deformation phenotype (Table 1). Therefore, we speculate that these 10 missense mutations are relatively severe loss-of-function alleles of *Notch*, whereas the other alleles are hypomorphic or context-dependent. We noticed that seven of these 10 mutations affect cysteine residues that form disulfide bonds and most of them are clustered in EGF-like repeats 8–10 (Table 1). Thus, Notch may be particularly sensitive to disruption of the basic structure of EGF-like repeats 8–10.

### 3.3. Notch Missense Mutations That Disrupted Intracellular Notch Trafficking

**Figure 4:**
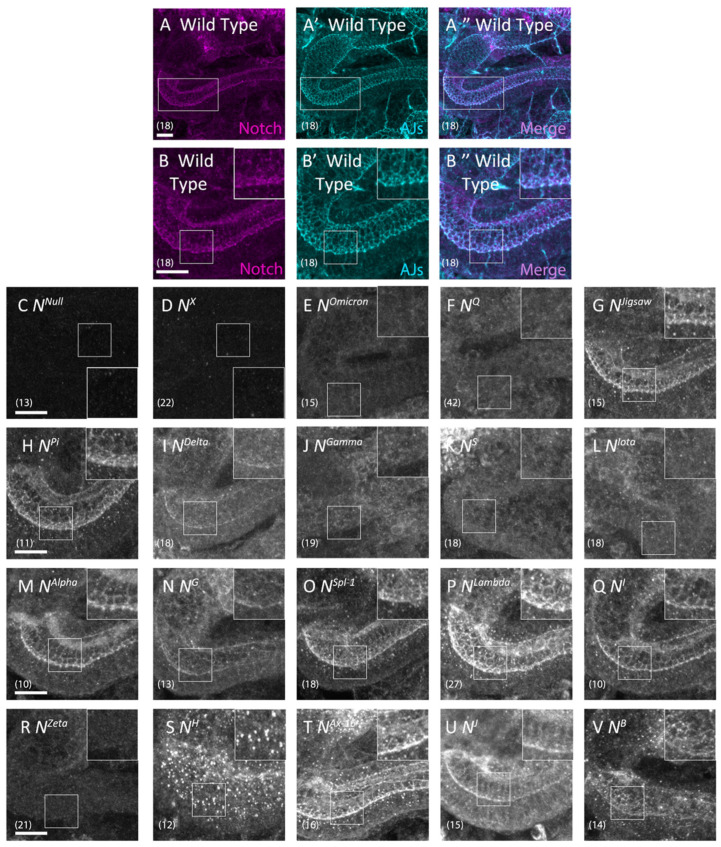
*Notch* alleles that disrupted intracellular Notch trafficking. (**A**,**A’’**,**B**,**B’’**) Notch and E-cadherin, a marker of adherens junctions (AJs), were detected in wild-type hindgut epithelium by anti-Notch (magenta in (**A**,**B**)) and anti-E-cadherin (turquoise in (**A’**,**B’**)) antibody staining. (**B**,**B’**,**B’’**) show high-magnification views of the regions outlined in (**A**,**A’**,**A’’**), respectively. Panels (**A’’**,**B’’**) are merged images of panels (**A**,**A’**,**B**,**B’**), respectively. (**C**–**V**) Notch was detected by anti-Notch antibody staining (white) in the hindgut epithelium of (**C**) *N*^55*e*11^, an amorphic allele of *Notch*; (**D**) *N^X^*, (**E**) *N^Omicron^*, (**F**) *N^Q^*, (**G**) *N^Jigsaw^*, (**H**) *N^Pi^,* (**I**) *N^Delta^*, (**J**) *N^Gamma^*, (**K**) *N^S^*, (**L**) *N^Iota^*, (**M**) *N^Alpha^*, (**N**) *N^G^*, (**O**) *N^Spl−^*^1^, (**P***) N^Lambda^*, (**Q**) *N^I^*, (**R**) *N^Zeta^*, (**S**) *N^H^*, (**T**) *N^Ax−^*^16^, (**U**) *N^J^*, and (**V**) *N^B^* hemizygotes. Insets are highly magnified images of regions outlined by white rectangles. The number of hindgut samples analyzed is shown in parentheses. Scale bars: 10 μm.

*Notch* mutations may disrupt Notch signaling by introducing defects in its folding or trafficking, with Notch consequently accumulating in the ER and/or endosomes [33]. An accumulation of Notch in the ER can, in turn, lead to the loss of Notch from AJs in epithelial cells. This loss is easily detected in the hindgut epithelium [31,33]. Thus, we analyzed the subcellular localization of Notch in the hindgut epithelium of embryo hemizygous for each of the 19 *Notch* missense mutations, and found abnormal intracellular distribution of Notch in 8 of the 19 mutants (Figure 4D–F,J–L,R,S; Table 1).

We compared signaling defects found in the central nervous system and boundary cells, assessed through *Notch* mutant phenotypes, with cellular defects related to Notch trafficking. We divided the *Notch* mutants accordingly into four classes based on the types of defects observed (Table 2), as follows: Class I comprised eight *Notch* mutants with normal Notch trafficking and normal Notch activity in the boundary cells and central nervous system. Class II comprised one *Notch* mutant that disrupted Notch trafficking but did not affect Notch activity in the boundary cells or nervous system. Class III comprised three *Notch* mutants that disrupted Notch activity in both the boundary cells and nervous system, but did not affect Notch trafficking. Class IV comprised seven *Notch* mutants that disrupted Notch trafficking and Notch activity in the boundary cells and nervous system. Based on these results, we conclude that a change in the amino acids in an EGF-like repeat can differ in its effect on Notch trafficking and activity, and that signaling defects and trafficking defects are not necessarily linked. Considering that amino acid substitutions in EGF-like repeats induced a range of defects in Notch trafficking and activities, the specific amino acid sequences within certain EGF-like repeats are likely crucial for normal Notch activity or trafficking [52].

### 3.4. Defects in Notch Trafficking and Loss of Notch Activity Were Not Always Coupled

**Figure 5:**
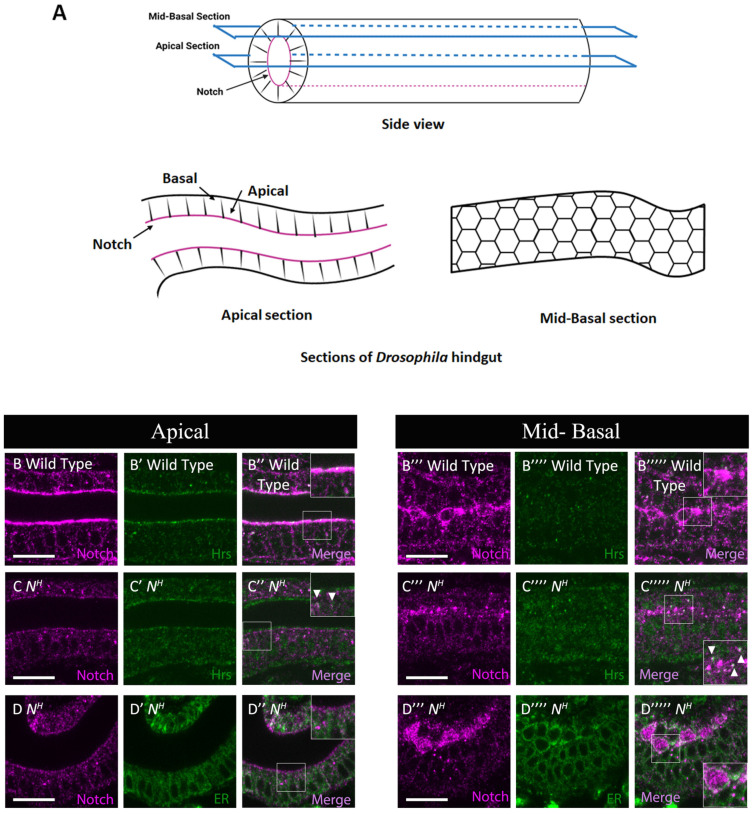
Notch accumulated abnormally in early endosomes of the hindgut epithelium in a Class II *Notch* mutant. (**A**) A diagram showing optical sections of the apical and mid-basal regions of the hindgut epithelium (Figures were made with the application from Biorender.com). The upper diagram shows the side view of the hindgut tube with apical and mid-basal sections, and the lower diagrams show the dorsal views of apical (left) and mid-basal (right) sections of the hindgut epithelium. The apical and mid-basal sections correspond to the microscopic images in (**B**–**D’’**) and (**B’’’**–**D’’’’’**), respectively, as indicated in the top of (**B**–**D’’’’’**). (**B**–**B’’’’’**) In wild-type hindgut epithelium, Notch (magenta) and Hrs (green), a marker of early endosomes, were stained with an anti-Notch (**B**,**B’’**,**B’’’**,**B’’’’’**) and anti-Hrs antibodies (**B’**,**B’’**,**B’’’’**,**B’’’’’**), respectively. (**C**–**D’’’’’**) Hindgut epithelium in the *N^H^* hemizygote, a Class II Notch mutant, stained for Notch (magenta in **C**,**C’’**,**C’’’**,**C’’’’’**,**D**,**D’’**,**D’’’**,**D’’’’’**), Hrs (green in **C’**,**C’’**,**C’’’’**,**C’’’’’**), and Pdi-GFP, an ER marker (green in **D’**,**D’’**,**D’’’’**,**D’’’’’**) were observed by anti-Notch, anti-Hrs, and anti-GFP antibody staining, respectively. Insets in (**B’’**,**B’’’’’**,**C’’**,**C’’’’’**,**D’’**,**D’’’’’**) are highly magnified images of regions outlined by white rectangles. White arrowheads point colocalized expression. All results were confirmed by staining biological triplicates. Scale bars: 10 μm.

The only Class II mutant in this study, *N^H^*, carries an amino acid substitution in the 29th EGF-like repeat with a cysteine (C) to serine (S) amino acid substitution at the 1155th amino acid residue (EGF-29, C1155S); this mutation affected the intracellular trafficking of Notch but not Notch function in lateral inhibition or inductive signaling in embryogenesis (Figure 2R, Figure 3R and Figure 4S). Notch was not detected in AJs in the hindgut of *N^H^* hemizygote embryos, where Notch is highly enriched in wild-type flies, but was instead found in punctate structures in the cytoplasm. To reveal the nature of such punctae, we analyzed the potential colocalization of Notch with markers of various intracellular compartments. We found that Notch colocalized with the early endosome marker Hrs (Hepatocyte growth factor regulated tyrosine kinase substrate) in the hindgut epithelium of *N^H^* hemizygote embryos (Figure 5C’’,C’’’’’) but not wild-type embryos (Figure 5B’’,B’’’’’; Table 1). On the other hand, Notch did not colocalize with markers for the ER (PDI-GFP) (Figure 5D’’,D’’’’’), *cis-*Golgi, recycling endosomes, or late endosomes under the same conditions (Figures S1–S5). Therefore, in *N^H^* hemizygotes, Notch is absent from AJs and accumulates in early endosomes in the hindgut epithelium, although such mislocalization of Notch does not appear to affect *Notch* signaling activity in this context. Under this condition, Notch presented at the plasma membrane appeared to be severely reduced, whereas the activity of Notch signaling was maintained normally. We speculated that this phenomenon can be explained by the nature of the *N^H^* mutation, which introduces an amino acid substitution in EGF-like repeat 29, included in the Abruptex domain [23]. Since mutations in the Abruptex domain often result in gain-of-function *Notch* alleles [23], it is possible that *N^H^* encodes a gain-of-function Notch while simultaneously reducing Notch presentation at the plasma membrane, which should reduce Notch signaling activity. Therefore, we speculate that a balance of these opposing effects on Notch activity belonging to the *N^H^* mutation may account for our observation that Notch signaling activity was normal in this mutant.

Conversely, our analyses revealed that the Class III alleles *N^Delta^* (EGF-9, D389N), *N^G^* (EGF-13, C535S), and *N^B^* (TMD, I1751K) showed attenuation in Notch activity in lateral inhibition, as predicted from brain deformation phenotype (Figure 2H,M,U) and in inductive signaling (Figure 3H,M,U) during embryogenesis, whereas Notch trafficking was normal in the hindgut epithelium (Figure 4I,N,V). These results suggest that the disruption of Notch activity is not always coupled with Notch trafficking defects. Considering the many factors that regulate Notch signaling at various layers within a cell, we speculate that these *Notch* missense mutations might disrupt some processes other than normal Notch trafficking. For example, *N^Delta^* and *N^G^* might disrupt ligand–receptor binding, since these mutations introduce amino acid substitutions into EGF-like repeats 9 and 13, respectively.

### 3.5. Notch Missense Mutations That Coupled Trafficking Defects with Loss of Notch Activity

**Figure 6:**
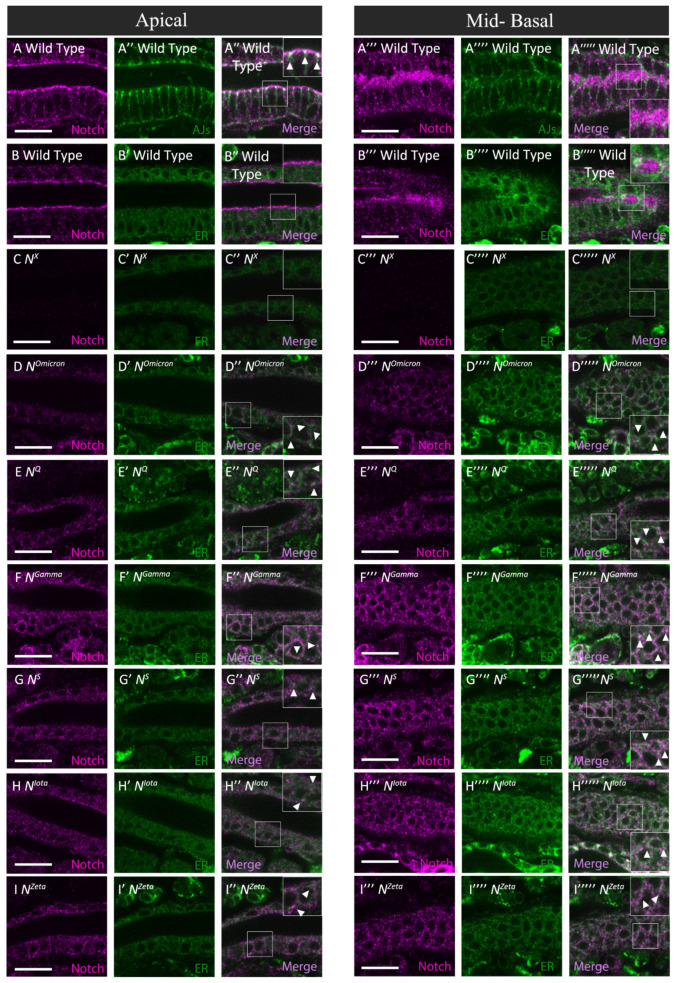
Notch accumulated abnormally in the ER of the hindgut epithelium in Class IV *Notch* mutants. (**A**–**I’’’’’**) Apical and mid-basal images corresponding to the diagrams of apical and mid-basal planes in Figure 5. (**A**–**B’’’’’**) Wild-type hindgut epithelium stained for Notch (magenta in **A**,**A’’**,**A’’’**,**A’’’’’**,**B**,**B’’**,**B’’’**,**B’’’’’**), E-Cadherin (green in **A’**,**A’’**,**A’’’’**,**A’’’’’**), and the ER marker Pdi-GFP (green in **B’**,**B’’**,**B’’’’**,**B’’’’’**) using anti-Notch, anti-E-Cadherin, and anti-GFP antibodies. (**C**–**I’’’’’**) Notch (magenta, left panels) and Pdi-GFP (green, middle panels) were observed by anti-Notch and anti-GFP antibody staining, respectively, in the hindgut epithelium of (**C**–**C’’’’’**) *N^X^*, (**D**–**D’’’’’**) *N^Omicron^*, (**E**–**E’’’’’**) *N^Q^*, (**F**–**F’’’’’**) *N^Gamma^*, (**G**–**G’’’’’**) *N^S^*, (**H**–**H’’’’’**) *N^iota^*, and (**I**–**I’’’’’**) *N^Zeta^* hemizygotes. Right-side panels in apical and mid-basal images, indicated by ’’ and ’’’’’, respectively, are merged from the left and middle images. Insets in the right panels indicated by ’’ and ’’’’’ are highly magnified views of regions in white rectangles. Intracellular punctae where Notch and Pdi-GFP colocalized are shown by white arrowheads. All results were confirmed by staining biological triplicates. Scale bars: 10 μm.

In total, 7 of the 19 *Notch* mutant alleles tested were Class IV, which exhibit trafficking defects and loss of Notch activity in both neural development and the formation of hindgut border cells (Figure 2C–E,I–K,Q; Figure 3C–E,I–K,Q; Table 1). The Class IV mutants include *N^X^* (EGF-8, C343S), *N^Omicron^* (EGF-8, C343Y), *N^Q^* (EGF-8, D331N), *N^Gamma^* (EGF-9, C398Y), *N^S^* (EGF-9, C407S), *N^Iota^* (EGF-10, C413S), and *N^Zeta^* (EGF-25, C993S). Notch was absent from AJs in all Class IV alleles (Figure 4D–F,J–L,R). Six of the seven Class IV mutants produced Notch proteins that accumulated in the ER, as shown by colocalization studies with Pdi-GFP (Figure 6D–I’’’’’), whereas hardly any Notch was detected in this organelle in wild-type embryos (Figure 6B’’,B’’’’’). On the other hand, Notch proteins derived from these six mutants did not colocalize with markers of other intracellular compartments, such as *cis*-Golgi, early endosomes, recycling endosomes, late endosomes, or of AJs (Figures S1–S5). Previous studies show that misfolded Notch protein was not transported to AJs because it was trapped in the ER [20,33]. These six mutants may also produce misfolded Notch that is not exported from the ER.

**Figure 7:**
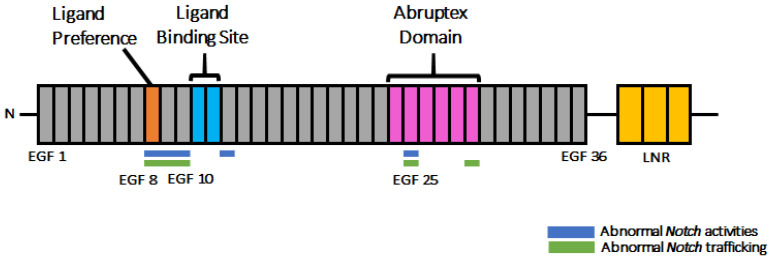
EGF-like repeats 8–10 and 25 are particularly sensitive to amino acid substitutions. A diagram showing EGF-like repeats with amino acid substitutions that disrupted Notch activity (blue bars) and Notch trafficking (green bars). The Abruptex domain (magenta), ligand-preference site (brown), and ligand-binding site (light blue) are also indicated. LNR repeats are shown in yellow.

Importantly, we found that six of the seven Class IV mutants have amino acid substitutions in EGF-like repeats 8–10. Thus, the EGF-like repeats in this region may be especially sensitive to structural perturbations (Figure 7). We speculate that these three EGF-like repeats may be particularly important in folding the whole extracellular domain of Notch.

The Class IV mutant *N^Zeta^*, which has an amino acid substitution in EGF-like repeat 25, accumulates Notch in the ER, suggesting that the mutation induces a severely misfolded product. EGF-like repeat 25 is a part of the Abruptex domain (EGF-like repeats 24–29) [21]. Amino acid substitutions within the Abruptex domain are known to induce gain-of-function mutations of *Notch*, suggesting that the Abruptex domain is involved in suppressing *Notch* activation [21]. It has also been suggested that the Abruptex domain contributes to forming Notch dimer proteins [53]. Given the apparent sensitivity of EGF-like repeat 25 to structural perturbation (Figure 7), the Abruptex domain may also be involved in the high-order organization of EGF-like repeats.

### 3.6. Disrupting Conserved Disulfide Bonds in Different EGF-like Repeats Induced Distinct Defects in Notch Activity and Trafficking

Although we observed different phenotypes associated with amino acid substitutions in individual EGF-like repeats, some differences may depend on the specific amino acids that replace the original residue rather than the position of the repeat. Four of the *Notch* missense mutants tested here—*N^X^* (EGF-8, C343S), *N^G^* (EGF-13, C535S), *N^Zeta^* (EGF-25, C993S), and *N^H^* (EGF-29, C1155S)—have the same amino acid substitution at the conserved second cysteine though occurring in different EGF-like repeats, and these cysteines were replaced with serine residues. Considering the differences in the behavior of these variants in our assay system, our data argue that, at least among the mutants we tested, the matter of which EGF-like repeat contains the mutation has important biological consequences (Figure 7). These results also suggest that our analysis of the defects induced in the various mutants also indicate, at least to some degree, a specific function of the EGF-like repeats containing the amino acid substitutions. On the other hand, our analyses also revealed that the *N^G^* (EGF-13, C535S) and *N^J^* (EGF-34, C1341Y) mutants, which have amino acid substitutions at conserved cysteines, did not accumulate Notch in the ER (Figure 4). This observation suggests that these EGF-like repeats are tolerant to structural perturbation with consequent misfolding. This also supports our idea that each EGF-like repeat plays specific roles in Notch folding.

## 4. Discussion

Notch has 36 EGF-like repeats in its extracellular domain [1]. Although these EGF-like repeats share a conserved structure, they play diverse roles as individual repeats and as clusters [3]. For example, EGF-like repeats 11–12 form the core ligand-binding site [54]. EGF-like repeats 10–12, 11–12, and 8 specifically contribute to *cis*-inhibition [55], *trans*-activation [9], and ligand selection [24], respectively. Genetic evidence suggests that EGF-like repeats 24–29, designated as the Abruptex domain, negatively regulate the Notch receptor [21,22,23]. However, relatively little is known about the specific roles of individual EGF-like repeats, and a complete high-order structure of Notch and its 36 EGF-like repeats in action has not been solved through structural analysis. In this study, we attempted to reveal the specific contributions of each EGF-like repeat to the activity, folding, and intracellular trafficking of Notch by studying the effect of missense mutations.

We analyzed 19 *Notch* mutants carrying missense mutations that were identified through a recent forward genetic screen [24] or as classic alleles. These mutations introduce unique amino acid substitutions into EGF-like repeats in 18 cases, and into the transmembrane domain in one case [24]. The mutants collected through genetic screening were isolated by clinical observation of Notch-related phenotypes in the wing or mechanosensory bristles [24]. To further characterize these mutants, we examined two other well-studied Notch-related phenotypes in embryonic tissues: lateral inhibition during central nervous system development and inductive signaling during boundary cell formation in the hindgut (Table 1). Our comparative analyses revealed that 10 out of 19 alleles exhibited either a neurogenic or brain deformation phenotype and boundary cells abnormalities (Table 1). In all cases, these two defects were observed coincidently. Therefore, the behavior of each of these 10 missense mutations was the same for lateral inhibition and for inductive signaling during embryogenesis. Although context dependency in Notch signaling has been studied extensively, it is still difficult to explain how it operates differently in various tissues [56]. Clear differences and similarities in the behaviors of the *Notch* missense mutants observed in this study provide an excellent opportunity to understand the molecular mechanisms of context-dependent Notch signaling.

As summarized in Figure 7, our analysis revealed that the EGF-like repeats sensitive to the amino acid substitutions with respect to the depletion of *Notch* activity are found in two regions within the 36 EGF-like repeats. One of these regions is EGF-like repeats 8–10, as revealed in the *Notch* missense mutants *N^X^*, *N^Omicron^*, *N^Q^*, *N^Gamma^*, *N^S^*, and *N^Iota^*. Intriguingly, the importance of EGF-like repeats 8–10 agrees with previous findings. For example, *O*-fucose modifications on EGF-like repeats 8 and 12 in Notch1 engage the EGF-like repeat 3 and the C2 domain, respectively, of the Jagged1 ligand [16]. Moreover, EGF-like repeat 8 modulates ligand binding selectivity in *Drosophila* [24]. EGF-like repeats 8–10 of Notch1 are required for DLL1- and DLL4-induced Notch signaling [57]. The importance of EGF-like repeats 8–10 has also been shown by analyzing *O*-fucose glycan modifications. *O*-fucose glycan modifications in EGF-like repeats 8, 9, and 12 of *Drosophila* Notch and in EGF-like repeats 8 and 12 of Notch1 specifically play important roles in modulating Notch–ligand binding [18,19]. Collectively, these results highlight the importance of EGF-like repeats 8–10 in Notch functions.

Another sensitive region was found in the EGF-like repeat 25, although this region was identified based on only one *Notch* mutant, *N^zeta^*. This region overlaps with the Abruptex domain (EGF-like repeats 24–29), which is known to negatively regulate *Notch* activity [21]. Genetic interaction analysis suggests that the Abruptex domain can be divided into two different clusters—EGF-like repeats 24–25, known as “suppressor of *Notch*”, and EGF-like repeats 27–29, known as “enhancer of *Notch*” [23]. The precise molecular function of Abruptex domain is unknown, and it is not clear why the *N^zeta^* mutation found in this region leads to a loss-of-function rather than a gain-of-function *Notch* phenotype. A more detailed study of this mutation along with other *Abruptex* alleles of *Notch* will likely provide insights into this mysterious domain. In summary, these two missense-sensitive clusters of EGF-like repeats correspond well to the EGF-like repeats that have been shown to play specific roles in *Notch* functions.

Our results also revealed that of seven *Notch* mutants with an amino acid substitution in one of the sensitive clusters, six accumulated Notch abnormally in the ER of the hindgut epithelium. We found seven Class IV *Notch* mutations in this study—*N^X^*, *N^Omicron^*, *N^Q^*, *N^Gamma^*, *N^S^*, *N^Iota^*, and *N^Zeta^*, which disrupted Notch trafficking and Notch activity (Table 2). Notch misfolding is known to cause Notch to accumulate in the ER [33]. Therefore, we speculated that amino acid substitutions in the EGF-like repeats of the sensitive clusters may induce global misfolding of Notch, which prevents the export of Notch from the ER by quality control mechanisms [20,33]. On the other hand, in Class III mutants, including *N^Delta^*, *N^G^*, and *N^B^*, Notch trafficking was normal, although Notch activity was reduced. However, in Class III mutants, defects in neural development were observed only in the brain, but not in the other part of the central nervous system. Considering that all Class IV mutants showed neurogenic phenotype in the entire central nervous system, underlying defects in Notch signaling may be different between Class III and Class IV, although all of them showed defects in inductive Notch signaling, as judged by the disruption of boundary cell formation. It is known that the activation of Notch signaling requires several steps in addition to proper Notch folding, such as ligand binding and Notch processing. Therefore, we speculate that some of these other steps might be disrupted in the Class III mutants, which may also explain the difference of neuronal phenotypes between Class III and Class IV.

As a potential limitation of this study, one could argue that the type of amino acid substitution found in the *Notch* mutants might be more important than which EGF-like repeat is affected. However, our analysis of the missense mutations in the *Notch* mutants *N^X^*, *N^G^*, *N^Zeta^*, and *N^H^*, which introduce the same amino acid substitution in the conserved second cysteine to serine, but in different EGF-like repeats, argues that identical amino acid changes introduced into different EGF-like repeats can differ in effect. Therefore, despite the limitation in the number of *Notch* alleles used here, our analyses successfully demonstrate, at least to some extent, the specificity of individual EGF-like repeats in Notch folding and activity.

Based on the results of our study, we propose that the EGF-like repeats 8–10 and 25 are particularly susceptible to structural perturbation with consequent misfolding and inactivation of Notch. We speculate that the ER may monitor the folding of these particular EGF-like repeats more strictly than other repeats because of their critical roles in Notch receptor functions. This idea should provide insights for further studies of correlations between Notch structure and function, and may provide molecular handles to assist in the functional interpretation of the missense variants that are found in human Notch receptors and are linked to diverse genetic disorders or cancers.

## Figures and Tables

**Table 1:** *Notch* missense mutations and their defects.

No	Name	EGF-like Repeat ^A^	Mutation Position	Notch Activity			Notch Trafficking	Notch Localization ^B^
				Bristle Formation	Lateral Inhibition	Inductive Signaling		
1	*N^X^*	EGF 8	C343S (C_2_S)	Absent	Neurogenic	Depletion	Abnormal	Loss
2	*N* *^Omicron^*	EGF 8	C343Y (C_2_Y)	Absent	Neurogenic	Depletion	Abnormal	ER
3	*N^Q^*	EGF 8	D331N	Absent	Neurogenic	Depletion	Abnormal	ER
4	*N^Jigsaw^*	EGF 8	V361M	Normal	Normal	Normal	Normal	AJs
5	*N* *^Pi^*	EGF 9	D374G	Absent	Normal	Normal	Normal	AJs
6	*N* *^Delta^*	EGF 9	D389N	Absent	Brain deformation	Depletion	Normal	AJs
7	*N* *^Gamma^*	EGF 9	C398Y (C_5_Y)	Absent	Neurogenic	Depletion	Abnormal	ER
8	*N^S^*	EGF 9	C407S (C_6_S)	Absent	Neurogenic	Depletion	Abnormal	ER
9	*N* *^Iota^*	EGF 10	C413S (C_1_S)	Absent	Neurogenic	Depletion	Abnormal	ER
10	*N^Alpha^*	EGF 11	E452K	Absent	Normal	Normal	Normal	AJs
11	*N^G^*	EGF 13	C535S (C_2_S)	Absent	Brain deformation	Depletion	Normal	AJs
12	*N* *^Spl^* *^−^* ^1^	EGF 14	I578T	Reduced	Normal	Normal	Normal	AJs
13	*N* *^Lambda^*	EGF 16	G668R	Absent	Normal	Normal	Normal	AJs
14	*N^I^*	EGF 16	G671D	Absent	Normal	Normal	Normal	AJs
15	*N* *^Zeta^*	EGF 25	C993S (C_2_S)	Absent	Neurogenic	Depletion	Abnormal	ER
16	*N^H^*	EGF 29	C1155S (C_2_S)	Absent	Normal	Normal	Abnormal	Early endosomes
17	*N* *^Ax−^* ^16^	EGF 29	G1174A	Reduced	Normal	Normal	Normal	AJs
18	*N^J^*	EGF 34	C1341Y(C_1_Y)	Absent	Normal	Normal	Normal	AJs
19	*N^B^*	TMD	I1751K	Normal	Brain deformation	Abnormal Gaps	Normal	AJs

^A^ EGF-like repeats are numbered in order starting with the repeat closest to the N terminal (EGF 1). ^B^ AJs: adherens junction. ER: endoplasmic reticulum.

**Table 2:** *Notch* missense mutations classified by types of defects in Notch activity and trafficking.

Classes	Notch Activity in Neuron & Boundary Cell	Notch Trafficking	*Notch* Alleles
I	Normal	Normal	*N^Jigsaw^*, *N^Pi^*, *N^Alpha^*, *N^Spl−^*^1^, *N^Lambda^*, *N^I^*, *N^Ax−^*^16^, *N^J^*
II	Normal	Abnormal	*N^H^*
III	Abnormal	Normal	*N^Delta^*, *N^G^*, *N^B^*
IV	Abnormal	Abnormal	*N^X^*, *N^Omicron^*, *N^Q^*, *N^S^*, *N^Gamma^*, *N^Iota^*, *N^Zeta^*

## Data Availability

All data reported are included and represented in the manuscript.

## Supplementary Materials

The following supporting information can be downloaded at: https://www.mdpi.com/article/10.3390/biom12121752/s1, Figure S1. Notch localized to AJs in Class II and IV *Notch* mutants. Figure S2. Class II and IV *Notch* mutants did not accumulate Notch in *cis-*Golgi. Figure S3. Class IV *Notch* mutants did not accumulate Notch in early endosomes. Figure S4. Class II and IV *Notch* mutants did not accumulate Notch in late endosomes. Figure S5. Class II and Class IV *Notch* mutants did not accumulate Notch in recycling endosomes.
