# Supplementary material for: Notch Missense Mutations in Drosophila Reveal Functions of Specific EGF-like Repeats in Notch Folding, Trafficking, and Signaling
Source: Biomolecules. 2022 Nov 25;12(12):1752. doi: 10.3390/biom12121752 (PMC9775759; doi:10.3390/biom12121752)
Supplement: Supplementary file 1 [file biomolecules-12-01752-s001.zip › biomolecules-1845033 - Supplementary - new11.28.pdf]

Supplementary Materials

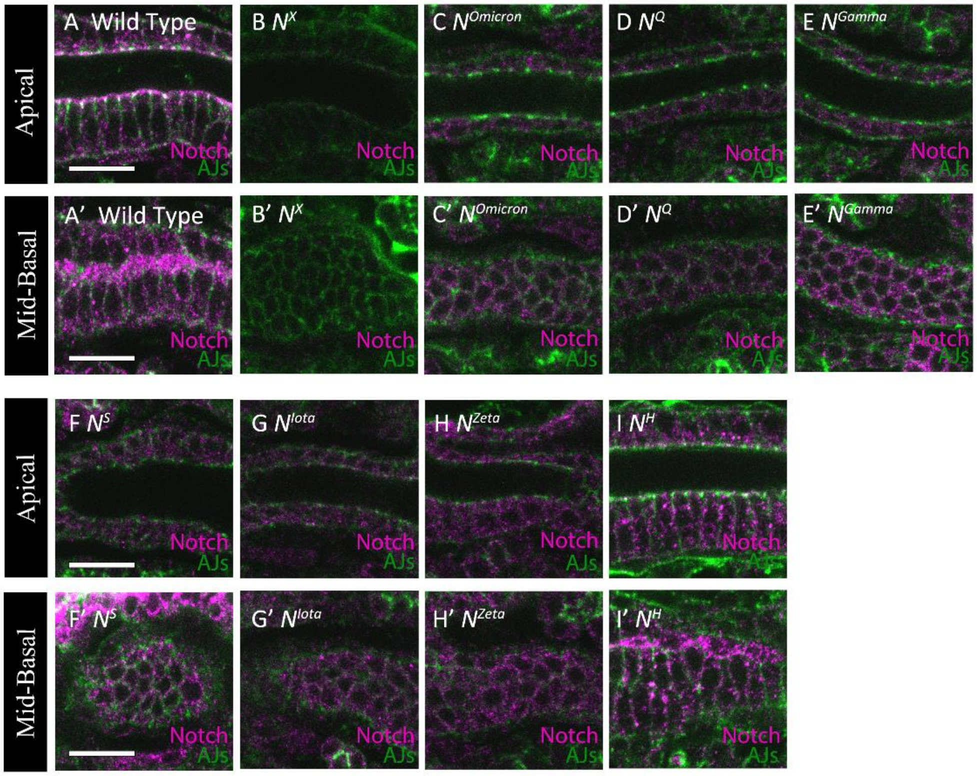

Figure S1. Notch did not localize to AJs in Class II and IV *Notch* mutants

(A-I') Notch (magenta) and adherens junctions (AJs; green) in hindgut epithelia in wild-type *Drosophila* (A and A') and in hemizygotes of N^X (B and B'), $N^{Omicron}$ (C and C'), N^Q (D and D'), N^{Gamma} (E and E'), N^S (F and F'), N^{Iota} (G and G'), N^{Zeta} (H and H'), and N^H (I and I'), stained by anti-Notch and anti-E-cadherin antibodies, respectively. Apical and mid-basal images (left side) correspond to the diagram of apical and mid-basal planes in Figure 5A. All results were confirmed by staining biological triplicates. Scale bars: 10 µm.

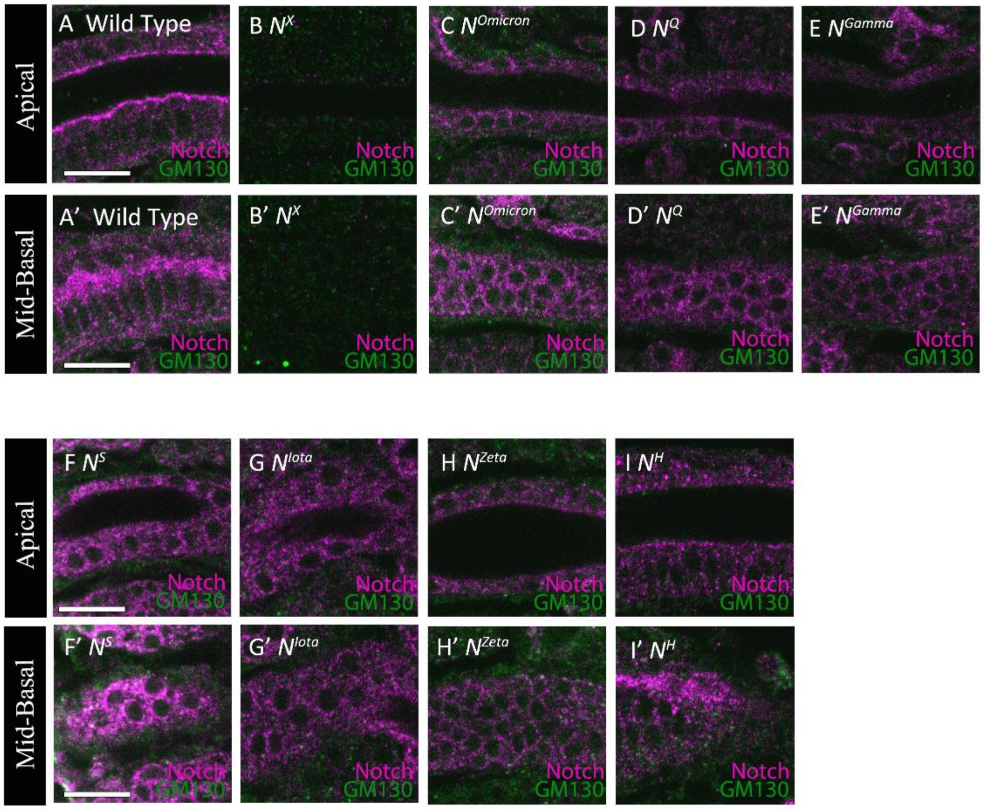

Figure S2. Class II and IV *Notch* mutants did not accumulate Notch in *cis*-Golgi.

(A-I') Notch (magenta) and *cis*-Golgi (green) of hindgut epithelia in wild type (A and A') and hemizygotes of N^{X} (B and B'), $N^{omicron}$ (C and C'), N^{Q} (D and D'), N^{Gamma} (E and E'), N^{S} (F and F'), N^{Iota} (G and G'), N^{Zeta} (H and H'), and N^{H} (I and I') were detected by anti-Notch and anti-GM130 antibody staining, respectively. Apical and mid-basal images (left side) correspond to the diagram of apical and mid-basal planes in Figure 5A. All results were confirmed through by staining of biological triplicates. Scale bars: 10 µm.

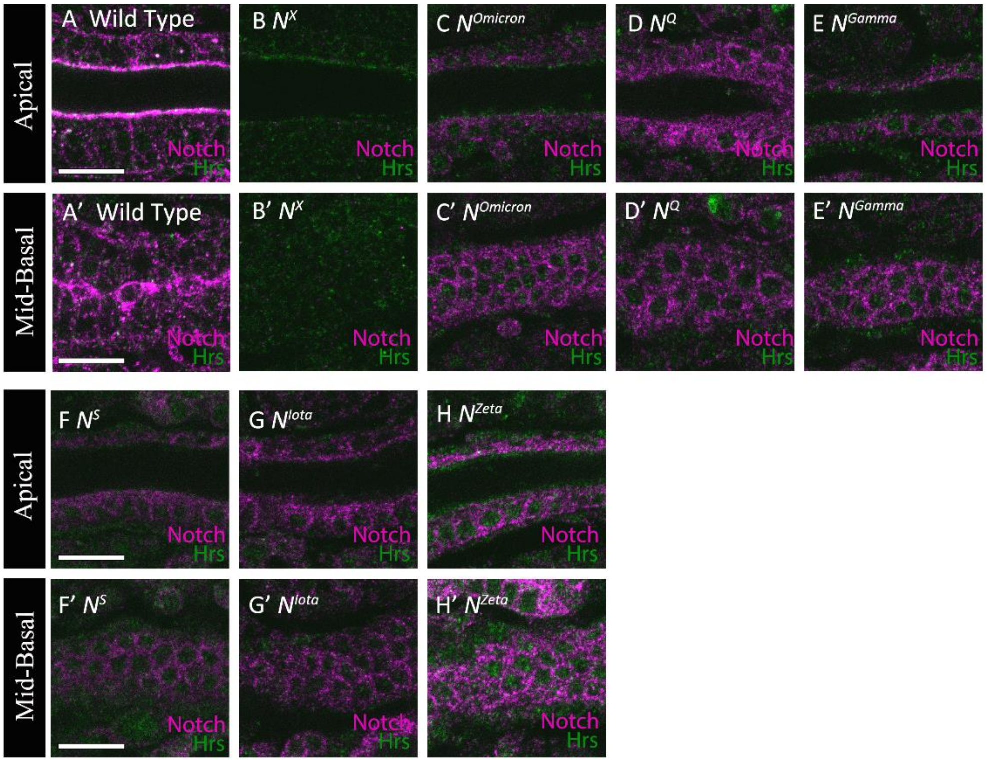

Figure S3. Class IV *Notch* mutants did not accumulate Notch in early endosomes

(A-H’) Notch (magenta) and early endosomes (green) of hindgut epithelia in wild type (A and A’) and in hemizygotes of N^X (B and B’), $N^{omicron}$ (C and C’), N^Q (D and D’), N^{Gamma} (E and E’), N^S (F and F’), N^{Iota} (G and G’), and N^{Zeta} (H and H’) were detected by anti-Notch and anti-Hrs antibody staining, respectively. Apical and mid-basal images left side) correspond to the diagram of apical and mid-basal planes in Figure 5A. All results were confirmed by staining biological triplicates. Scale bars: 10 μm.

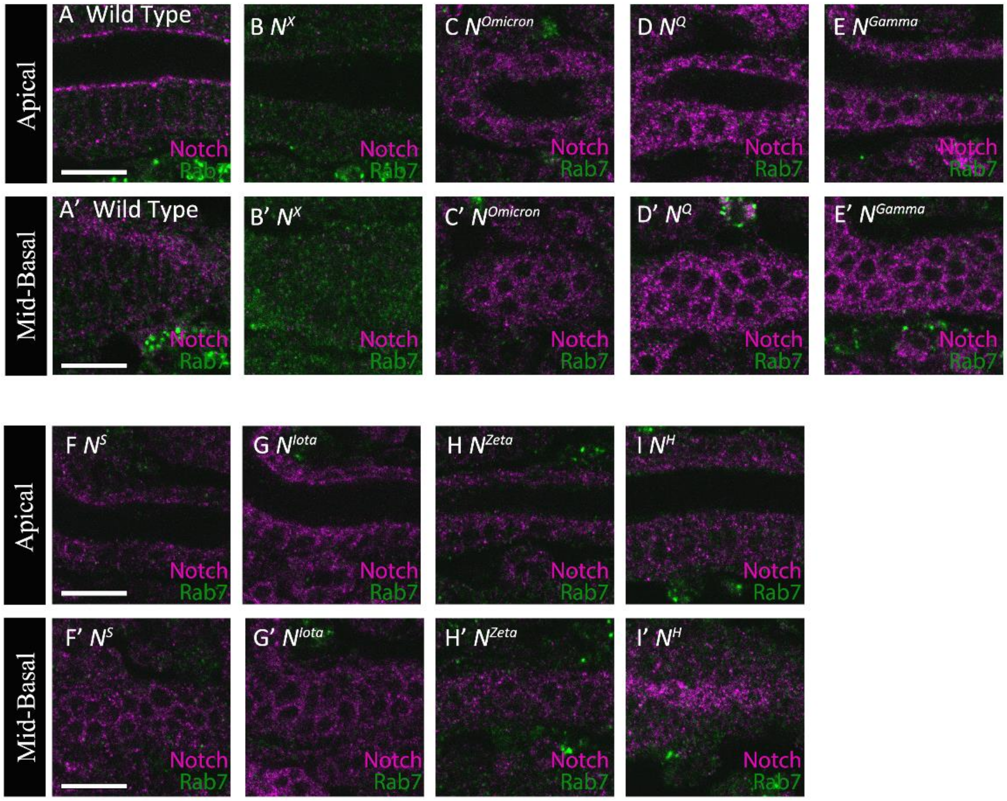

Figure S4. Class II and IV *Notch* mutants did not accumulate Notch in late endosomes

(A-I') Notch (magenta) and late endosomes (green) of hindgut epithelia in wild-type embryos (A and A') and hemizygotes of N^X (B and B'), $N^{omicron}$ (C and C'), N^Q (D and D'), N^{Gamma} (E and E'), N^S (F and F'), N^{Iota} (G and G'), N^{Zeta} (H and H'), and N^H (I and I'), detected with anti-Notch and anti-Rab7 antibody staining, respectively. Apical and mid-basal images (left side) correspond to the diagram of apical and mid-basal planes as described in Figure 5A. All results were confirmed by staining biological triplicates. Scale bars: 10 µm.

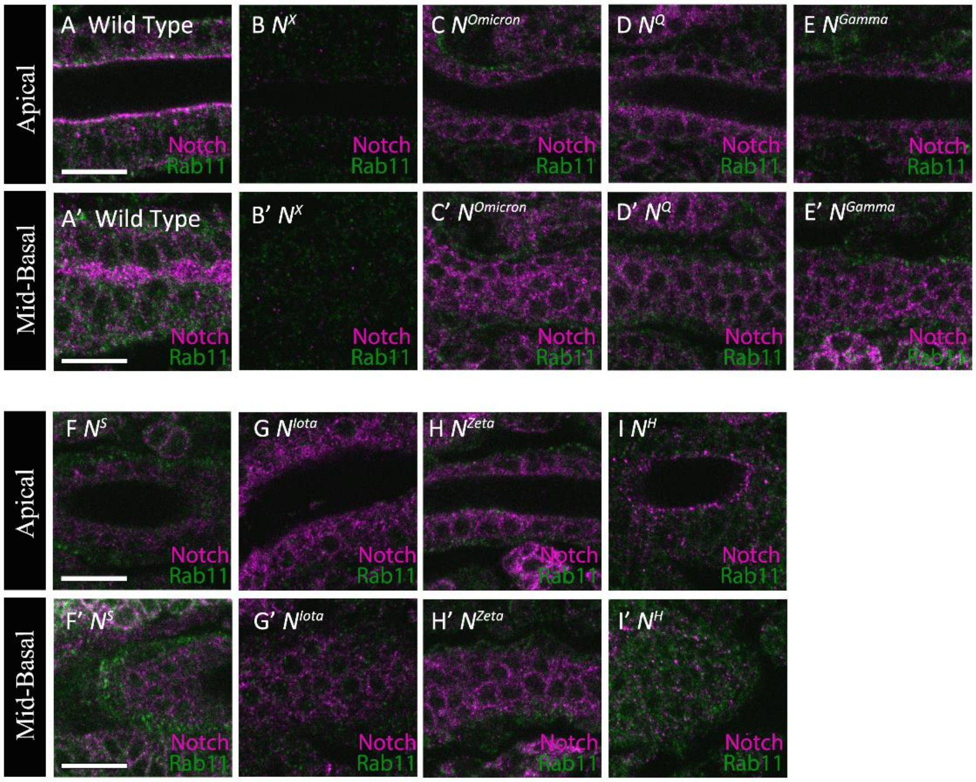

Figure S5. Class II and Class IV *Notch* mutants did not accumulate Notch in recycling endosomes (A-I') Notch (magenta) and recycling endosomes (green), detected by anti-Notch and anti-Rab11 antibody staining, respectively, in hindgut epithelia in wild-type embryos (A and A') and hemizygotes of N^X (B and B'), $N^{omicron}$ (C and C'), N^Q (D and D'), N^{Gamma} (E and E'), N^S (F and F'), N^{Iota} (G and G'), N^{Zeta} (H and H'), and N^H (I and I'. Apical and mid-basal images (left side) correspond to the diagram of optical planes in Figure 5A. Results were confirmed by staining biological triplicates. Scale bars: 10 µm.
